# Development and Evaluation of a Virtual Reality Puzzle Game to Decrease Food Intake: Randomized Controlled Trial

**DOI:** 10.2196/31747

**Published:** 2022-02-03

**Authors:** Yunxin Liu, Angelos Stamos, Siegfried Dewitte, Zeph M C van Berlo, Laura N van der Laan

**Affiliations:** 1 Department of Marketing and Retailing ESSCA School of Management Lyon France; 2 Department of Management, Society and Communication Copenhagen Business School Copenhagen Denmark; 3 Behavioral Economics and Engineering Group Faculty of Economics and Business KU Leuven Leuven Belgium; 4 Amsterdam School of Communication Research University of Amsterdam Amsterdam Netherlands; 5 Department of Communication and Cognition Tilburg University Tilburg Netherlands

**Keywords:** virtual reality, pre-exposure, self-control, hedonic consumption, food cravings

## Abstract

**Background:**

Virtual reality (VR) has gained popularity in daily life, and VR food cues seem to elicit food cravings, similar to real food cues. However, little is known about the impact of VR food cues on actual food intake.

**Objective:**

In real life (RL), exposure to food cues in a situation in which the desire to eat food interferes with the completion of a food-related task reduces the subsequent food intake (ie, the pre-exposure effect). In this study, we examine, on the one hand, whether the pre-exposure effect could be replicated in RL and, on the other hand, whether this effect could be extended to VR contexts.

**Methods:**

The study used a 2 (stimulus type: food vs nonfood) × 2 (mode: VR vs RL) between-subject design (n=175). Participants were randomly assigned to 1 of the 4 conditions.

**Results:**

We found the main effect of mode on food intake, with a higher food intake after both VR conditions than after RL conditions (*P*=.02). In addition, among female participants, we found that exposure to both food cues (ie, VR and RL) resulted in lower food intake than exposure to both nonfood cues (*P*=.05). In contrast, this effect was not observed among male participants (*P*=.34). Additionally, VR and RL cues generated similar emotional and behavioral responses (eg, arousal and game difficulty).

**Conclusions:**

We were unable to replicate the exposure effect in our complete sample. Subgroup analyses, however, showed that for women, exposure to food cues (either in VR or in RL) reduces food intake, indicating that a VR pre-exposure procedure may effectively be applied exclusively for women.

**Trial Registration:**

ClinicalTrials.gov NCT05169996; https://clinicaltrials.gov/ct2/show/NCT05169996

## Introduction

### Background

People are often said to *eat with their eyes* [1]. It is therefore not surprising that exposure to tempting foods has been linked to overeating and excessive food consumption in the past [2]. However, exposure to food temptations does not necessarily lead to higher food intake. Research has shown that when people perform a food-related task (eg, a puzzle-solving task with tempting candies), prior to being exposed to tempting foods, this can actually decrease (rather than increase) food intake [3]. This mechanism, often referred to as the *pre-exposure effect*, was first described by Geyskens et al [4] and has since shown to be effective across cultures [5] and for children [6]. These diverse applications have made it a promising candidate effect to be turned into a behavioral change tool. However, the potential of implementing it as a behavioral change tool is relatively low because it seems to require real temptations to be effective [4]. Given that tempting foods may become perishable after a certain period and that upon being touched, the food needs to be thrown away, the usage of the pre-exposure procedure as a behavioral change tool can be costly and logistically inconvenient.

Recently, technological advances, such as virtual reality (VR), have made it possible to run the pre-exposure procedure in a cost-efficient and flexible way. VR can simulate a virtual environment with tempting food stimuli by delivering multisensory cues [7], which leads to a strong sense of physical presence in an immersive environment [8]. In other words, in the context of computer-generated artificial content, VR has made it easier for consumers to interact with tempting foods in an immersive environment. Research in the food domain has shown that VR food cues elicit similar emotional, psychological, and behavioral responses as compared to those produced by real food cues [9-11]. These studies have mainly focused on the relationship between VR food cues and food cravings, which is defined as “an intense desire to eat a specific food that is difficult to resist” [12]. Yet, little is currently known about the impact of VR food cues on actual food intake. Given that VR continues to grow in popularity [13], gaining a deeper understanding of how foods presented in VR could influence subsequent real food intake is becoming more important. Therefore, with this study, we seek to shed new light on the impact of VR on food intake and particularly examine how food cues can be used to stimulate self-regulation in food intake.

### The Pre-exposure Procedure

The pre-exposure procedure is a 2-phase paradigm. In the original study that identified this procedure, participants in the experimental condition performed a consumer knowledge task in which they were asked to associate various wrappers of candies with the corresponding flavors [4]. In contrast, participants in the control condition were asked to link various colors to the corresponding concepts (eg, green with grass). Following that, participants engaged in a taste test of a similar tempting food (eg, chocolate candies), which was presented as a different study. The basic finding of this procedure is that exposure to physical temptations results in lower subsequent food intake than exposure to nontemptations. The pre-exposure procedure has also been replicated with other food-related tasks, such as a word formation task [14,15] and a puzzle-solving task [3]. The assumed mechanism accounting for the pre-exposure effect is that participants experience a behavioral conflict between food desirability (ie, food cravings) and the engagement of food-related tasks in the first phase [6]. In other words, participants engage in self-control efforts to curb food cravings in this phase [16]. When facing a similar tempting situation in the second phase (ie, a taste test), these participants exert similar self-control efforts in response to temptations. Florack et al [17] did not replicate the pre-exposure effect, but as their participants were under 6 years of age, this failure to replicate did not necessarily rule out the mechanism of coping with behavioral conflict, as self-control abilities are not fully developed among younger children [17].

In sum, the first aim of this paper is to replicate the pre-exposure effect with real food temptations among adults. We posit that exposure to real tempting food cues decreases subsequent intake of a similar tempting food as compared to exposure to nonfood cues. Moreover, the aim of the research is not only to replicate the pre-exposure effect with real foods but more importantly to better understand the potential of VR in the pre-exposure procedure. The next sections will focus on the extension of the pre-exposure effect to VR contexts.

### Responses to VR Food Cues

There is a large body of research on how consumers respond to food cues in VR. Prior research has shown that VR food cues can produce emotional (eg, anxiety and arousal) or behavioral (eg, product selection) responses similar to those observed in real-life (RL) contexts [10,11,18,19]. Previous research has also focused on the relationship between VR food cues and food cravings [7,12,20-22]. For instance, food cravings triggered by VR food cues were significantly higher than those produced by VR neutral cues (ie, as a baseline condition) but were lower than those produced by real food [7]. In a similar vein, exposure to food cues induced stronger food cravings than exposure to nonfood cues in both VR and RL contexts; note that the difference was weaker in VR conditions than in RL conditions [22]. Additionally, exposure to hedonic food cues (eg, pizza) provoked high levels of food cravings than exposure to utilitarian food cues (eg, salad) in VR contexts [12,21]. The latter findings seem relevant to this research because the pre-exposure procedure has primarily focused on the intake of hedonic food. It should be noted that prior research mainly focused on the effect of VR cues on food cravings instead of food intake. To the best of our knowledge, only 1 study investigated how VR food cues affect food intake [23]; however, the authors focused on the difference in food intake between different eating environments (restaurant vs common room). This research fills this gap by examining the effect of food cues on food intake in both VR and RL contexts.

Overall, the prior literature suggests that consumers’ emotional and behavioral responses in food-related VR contexts are similar (albeit weaker) to those in the RL [7,22]. In addition, VR-based food cues can produce higher levels of food cravings than VR-based nonfood cues. As mentioned earlier, an important assumption of the observed pre-exposure effect is that the food presented in the pre-exposure phase should be tempting and elicit a desire to eat. Only if the food is tempting will the desire to eat the tempting food interfere with the completion of a food-related task in RL contexts, which further reduces subsequent food intake. Could food cravings triggered by VR food cues produce a similar effect? The prior literature suggests that foods presented in VR are sufficiently lifelike to elicit such feelings of craving [22]. Therefore, we expect that the pre-exposure effect could be observed in the VR contexts as well.

### Aims of This Study

In sum, although there is a burgeoning body of research that focuses on how VR affects food cravings, marketers and researchers are struggling to fully understand the impact of VR on eating behaviors (eg, food intake). It is important to note that our research focuses on the actual food intake (instead of food cravings) after interacting with food or nonfood cues in both VR and RL contexts. For the application in the VR context, we designed a task context where people could interact with the food in VR. To validate that this procedure could be used to trigger the pre-exposure effect, we tested it both in an RL and in a VR context. We assume that participants’ behavior in the VR context will be similar to their behavior after exposure to physical food temptations. In addition, prior research has shown that passive exposure to VR food cues induces high levels of food cravings as compared to VR nonfood cues [22]. Therefore, we assume that food cravings induced by VR food cues interfere with the completion of a food-related task (ie, puzzle game) in the exposure phase. Together, we expect that interaction with VR food cues in a pre-exposure paradigm decreases subsequent intake of a similar tempting food as compared to exposure to VR nonfood cues.

## Methods

### Design and Participants

This study used a 2 (stimulus type: food vs nonfood) × 2 (mode: VR vs RL) between-subject design. Participants were randomly assigned to 1 of the 4 conditions. A total of 218 participants (18-30 years old) were recruited with flyers and posters from a large Western European university. Participants received course credits or €7.50 for participation. This study was approved by the university ethical committee of the institution (file no. 2018-PC-9033) where the corresponding author was employed at the time of data collection. All participants provided written informed consent. In addition to the 4 conditions reported in this paper (n=175), an additional condition (branded VR, n=43) was collected with the aim to investigate the effect of brands presented in VR on brand memory and purchase intention, and the results are reported elsewhere [24,25].

### Procedure

Participants were asked to refrain from eating 2 hours before the study. After entering the laboratory, participants were told that they were participating in 2 unrelated studies: a puzzle game and a taste test. First, they were asked to finish a tangram (puzzle game) with either food products (ie, pieces of chocolate) or nonfood products (ie, pieces of wood), either in VR or in RL (depending on their condition). Following that, they were asked to participate in a taste test of chocolate candies. Given the taste test, it was required that the participants were not allergic to peanuts (self-reported). Finally, they completed a questionnaire measuring game experiences (ie, entertainment and difficulty of the puzzle game) and emotional responses (ie, arousal and valence). We also measured the attractiveness of chocolate and the desire to eat chocolate for participants in the food cues condition (both VR and RL contexts). Participants also reported their demographic data (eg, age and gender) and data on height and weight. In addition, participants’ hunger levels and the completion time of the puzzle game were measured as covariates. After all measurements, the participants were thanked for their participation and debriefed on the fact that both studies were related. Note that the groups that were in the RL conditions also got an opportunity to play the VR game at the end of the procedure after all measurements (as the study was advertised as a study involving VR and the participants were told in the factsheet that they would engage in a VR game during participation).

### Stimulus Materials

**Figure 1:**
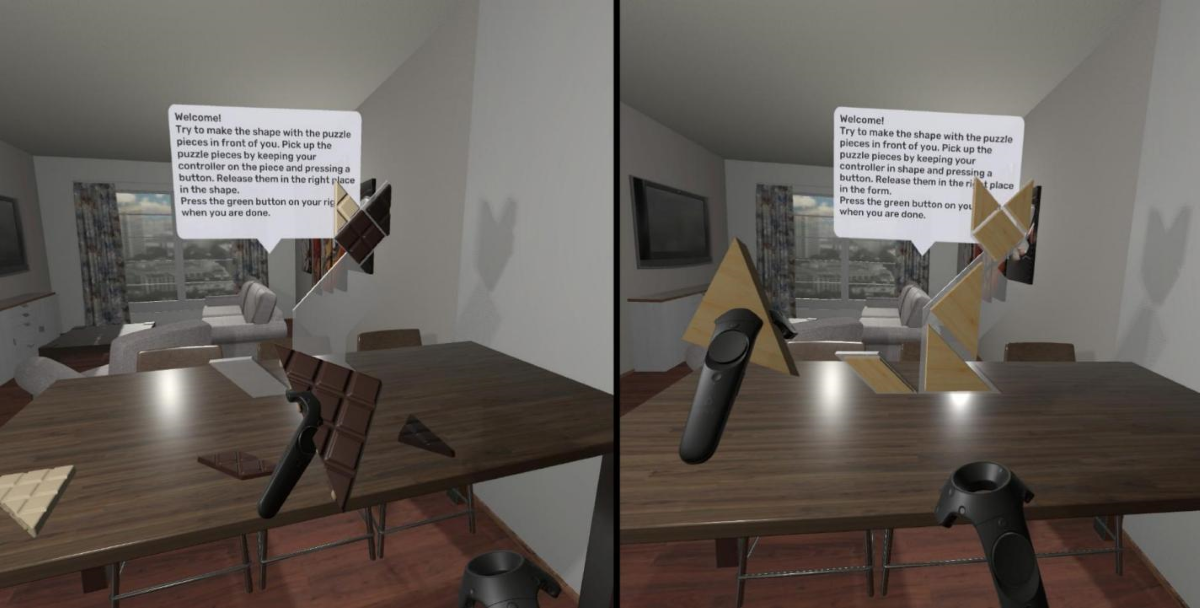
Screenshot of VR puzzle game with chocolate (left panel; VR food condition) and wooden (right panel; VR nonfood condition) puzzle pieces. Participants could pick up puzzle pieces with the controller and place them in the outline in front of them.

An immersive VR game was developed with a gameplay based on the pre-exposure effect. The game is played by wearing a head-mounted display VR (HMD-VR; HTC Vive) instrument and players can interact in the virtual environment with handheld controllers in the lab. The task in the game is to finish a tangram puzzle. Two versions of the game were developed, one in which the tangram pieces were tempting food products (ie, pieces of chocolate) and the other in which the tangram pieces were plain pieces (see Figure 1). Players have to physically move the tangram pieces with the grab button on the controller and put them together. In total, participants were asked to complete 3 levels: in each of the levels, they had to puzzle a particular shape (eg, cat, house, and dog). A regular wooden tangram game was used in the nonfood product, non-VR condition, while pieces of real chocolate were used in the food product, non-VR condition (Multimedia Appendix 1). Chocolate is generally seen as a highly tempting food and can elicit a desire to eat [4,6,16]. In addition, VR chocolates elicit stronger food cravings than VR nonfood [22]. A conceptually similar task (forming a word with gummy bears) was shown to be effective for inducing the pre-exposure effect [14].

### Measures

#### Food Intake

In the taste test, participants were presented with 2 bowls of chocolate, one with chocolate-covered peanuts from the brand M&M’s and the other with private label chocolate-covered peanuts. Participants were instructed to taste at least 1 of each bowl and were allowed to eat as much of the chocolate as needed to evaluate the products on several dimensions (eg, “Are they crunchy” and “Do they have an intense flavor?”). Food intake was measured by weighing the bowls before and after the test. The participants were left alone in the lab for 5 minutes during the taste test to avoid socially desirable behavior. The distribution of food intake was skewed, so we transformed the food intake with a logarithmic format.

#### Game Experiences

We measured the perceived entertainment and difficulty of puzzle games as indicators of game experiences. Specifically, the perceived entertainment was measured with 4 items (eg, “Playing the game has been exciting” and “I have had fun playing the game”) on a 7-point scale [26]. The perceived difficulty was measured with 4 items (eg, “To what extent did you find the game easy” and “How well do you think you performed in the game”) on a 7-point scale. In the study, both measures were reliable (entertainment: Cronbach α=.86; difficulty: Cronbach α=.71).

#### Emotional Responses

To assess whether emotional responses induced in VR contexts were similar to those generated in RL contexts, we measured arousal and valence using the Self-Assessment Manikin (SAM) scale [27]. The SAM scale was designed to measure the emotional state with a row of 5 nonverbal and graphic manikins that differ in levels of 2 affective dimensions (eg, valence and arousal). For the valence measure, the SAM scale ranges from a happy and smiling figure to an unhappy and frowning figure. For the arousal measure, the SAM scale ranges from an excited figure to a relaxed figure. Participants can indicate their current emotional state on any of the 5 figures or between any 2 figures. In other words, participants were asked to report their emotional state after playing the puzzle game on a 9-point scale.

#### The Appeal of Tempting Foods

In the food cues condition, we also measured the appeal of food. Specifically, the attractiveness was measured with a single item (ie, “How appealing was the chocolate you saw while playing the game?”) on a visual analog scale (VAS) ranging from 0 (“not at all”) to 100 (“a whole lot”). Similarly, the desire to eat chocolate was measured with a single item (ie, “How much did you feel like eating the chocolate?”) on a 7-point scale ranging from 1 (“not at all”) to 7 (“a whole lot”). The 2 measures were correlated with each other (*r*=0.75, *P*<.001).

## Results

### Data Cleaning

Data from 13 participants were excluded from analysis because of nonconforming to the study tasks (ie, refusing to eat or to eat less than 2 chocolate-covered peanuts [4 g] in the bogus taste task, n=4), impossible values (ie, eating more than 200 g of chocolate-covered peanuts in the bogus taste task, n=1), reporting a low preference for chocolate (ie, a score of 2 or less on a 7-point scale ranging from 1 for “not at all” to 7 for “a lot” for the question “How much do you like chocolate?”, n=7), or spontaneous mention of not liking the peanut M&M’s (n=1). The final sample consisted of 162 adults (118 [72.8%] females, 44 [27.2%] males) with a mean age of 22.4 years (SD 4.1) and a mean body mass index (BMI) of 21.9 kg/m^2^ (SD 2.7). Table 1 presents the characteristics of participants for the 4 conditions; the conditions did not differ significantly on any of the characteristics.

**Table 1:** Characteristics of the study population (N=162).

Characteristic	Real food (n=42)	Real nonfood (n=41)	Virtual food (n=40)	Virtual nonfood (n=39)	Difference between conditions
Age (years), mean (SD)	22.55 (5.11)	22.16 (3.30)	22.13 (4.46)	22.54 (3.50)	*F*_3,158_=0.092, *P*=.97
BMI^a^ (kg/m^2^), mean (SD)	21.72 (2.58)	22.46 (3.53)	21.50 (1.98)	22.10 (2.35)	*F*_3,158_=1.010, *P*=.39
Female participants, %	81.0	78.1	70.0	61.5	*χ*^2^_3_=4.612, *P*=.20
Weight concerns^b^, mean (SD)	5.23 (2.22)	5.13 (2.03)	5.43 (2.31)	5.51 (2.13)	*F*_3,158_=0.240, *P*=.87
Chocolate preference^c^, mean (SD)	6.07 (1.00)	5.66 (1.35)	5.80 (1.29)	5.77 (1.33)	*F*_3,158_=0.823, *P*=.48
Hunger^d^, mean (SD)	51.10 (23.29)	52.13 (25.21)	56.78 (25.38)	52.80 (23.28)	*F*_3,157_=0.423, *P*=.74
Time since last intake (minutes), mean (SD)	164.86 (72.09)	203.08 (148.70)	189.80 (136.39)	175.54 (120.85)	*F*_3, 157_=.754, *P*=.52

^a^BMI: body mass index.

^b^The question “To what extent are you concerned with your weight?” answered on a 9-point scale ranging from 1 (“not at all”) to 9 (“completely”).

^c^The question “How much do you like chocolate?” answered on a 9-point scale ranging from 1 (“not at all”) to 7 (“a lot”).

^d^The question “How hungry are you right now?” answered on a visual analog scale (VAS) scale ranging from 0 (“not hungry at all”) to 100 (“very hungry”).

### Randomization Check

We conducted a randomization check to test whether the sample was distributed equally across conditions (see Table 1). We compared the difference between different conditions on age (*F*_3,158_=0.092, *P*=.97), gender (*χ^2^*_3_=4.612, *P*=.20), BMI (*F*_3,158_=1.010, *P*=.39), weight concerns (*F*_3,158_=0.240, *P*=.87), chocolate preference (*F*_3,158_=0.823, *P*=.48), hunger (*F*_3,157_=0.423, *P*=.74), and time since last intake (*F*_3,157_=0.754, *P*=.52). These results indicate that our randomization was successful.

### Effects of Stimulus Type and Mode of Pre-exposure on Food Intake

ANOVA with stimulus type (food vs nonfood) and mode (VR vs RL) as independent variables and food intake (natural log transformed) as the dependent variable was performed. No significant main effect of stimulus type was found (*F*_1,158_=2.234, *P*=.14, partial η^2^=0.014). A main effect of mode was found (*F*_1,158_=5.556, *P*=.02, partial η^2^=0.034). Food intake (g) was higher in the VR condition (log-transformed M=3.202, SD 0.564; inverse-log-transformed M=24.581, SD 1.758) than in the RL condition (log-transformed M=2.990, SD 0.580; inverse-log-transformed M=19.886, SD 1.786). The interaction between stimulus type and mode was not significant (*F*_1,158_=0.004, *P*=.95, partial η^2^<0.001). R^2^ of the complete model was 0.047 (adjusted R^2^=0.029); we also performed an analysis controlling for the hunger level, liking of chocolate candies, and time since the last intake as covariates, and the significant level of the main effects was not substantially different.

### Effects of Stimulus Type and Mode of Pre-exposure on Food Intake Separated by Gender

As previous studies have shown that pre-exposure effects are in some instances specific to males [15] or females [6], we explored this factor in this study as well. We tested the effect of stimulus type and mode of pre-exposure on food intake for females and males, respectively.

#### Females

ANOVA with stimulus type (food vs nonfood) and mode (VR vs RL) as independent variables and food intake (natural log transformed) as the dependent variable was performed. A significant main effect of stimulus type was found (*F*_1,114_=3.986, *P*=.05, partial η^2^=0.034). Food intake (g) was higher in the nonfood condition (log-transformed M=3.108, SD 0.530; inverse-log-transformed M=22.376, SD 1.699) compared to the food condition (log-transformed M=2.902, SD 0.595; inverse-log-transformed M=18.211, SD 0.813). A main effect of mode was found (*F*_1,114_=4.478, *P*=.04, partial η^2^=0.038). Food intake (g) was higher in the VR condition (log-transformed M=3.121, S*D* 0.568; inverse-log-transformed M=22.669, SD 1.765) than in the RL condition (log-transformed M=2.904, SD 0.562; inverse-log-transformed M=18.247, SD 1.754). The interaction between stimulus type and mode was not significant (*F*_1,114_=0.052, *P*=.82, partial η^2^<0.001). R^2^ of the complete model was 0.070 (adjusted R^2^=0.045).

#### Males

ANOVA with stimulus type (food vs nonfood) and mode (VR vs RL) as independent variables and food intake (natural log transformed) as the dependent variable was performed. No significant main effect of stimulus type was found (*F*_1,40_=0.947, *P*=.34, partial η^2^=0.023). No significant main effect of mode was found (*F*_1,40_=0.025, *P*=.87, partial η^2^=0.001). The interaction between stimulus type and mode was not significant (*F*_1,40_=0.927, *P*=.34, partial η^2^=0.023). R^2^ of the complete model was 0.038 (adjusted R^2^=–0.034).

### Effects of Stimulus Type and Mode of Pre-exposure on Indicators of Game Experience (Entertainment, Difficulty)

To test whether the game experience produced by VR cues was similar to that produced by RL cues, ANOVA with stimulus type (food vs nonfood) and mode (VR vs RL) as independent variables and entertainment as the dependent variable was performed. No significant main effect of stimulus type was found (*F*_1,158_=0.001, *P*=.98, partial η^2^<0.001). No main effect of mode was found (*F*_1,158_=0.045, *P*=.83, partial η^2^<0.001). The interaction between stimulus type and mode was not significant (*F*_1,158_<0.001, *P*=.99, partial η^2^<0.001). R^2^ of the complete model was <0.001 (adjusted R^2^=0.019).

ANOVA with stimulus type (food vs nonfood) and mode (VR vs RL) as independent variables and difficulty as the dependent variable was performed. No significant main effect of stimulus type was found (*F*_1,158_=1.588, *P*=.21, partial η^2^=0.010). No main effect of mode was found (*F*_1,158_=0.398, *P*=.53, partial η^2^=0.003). The interaction between stimulus type and mode was not significant (*F*_1,158_=2.952, *P*=.09, partial η^2^=0.018). R^2^ of the complete model was 0.031 (adjusted R^2^=0.012).

### Effects of Stimulus Type and Mode of Pre-exposure on Emotions (Valence, Arousal)

To test whether emotional responses produced by VR cues are similar to those produced by RL cues, ANOVA with stimulus type (food vs nonfood) and mode (VR vs RL) as independent variables and valence as the dependent variable was performed. No significant main effect of stimulus type was found (*F*_1,158_=0.791, *P*=.38, partial η^2^=0.005). No main effect of mode was found (*F*_1,158_=0.281, *P*=.60, partial η^2^=0.002). The interaction between stimulus type and mode was not significant (*F*_1,158_=0.849, *P*=.36, partial η^2^=0.005). R^2^ of the complete model was 0.012 (adjusted R^2^=–0.007).

ANOVA with stimulus type (food vs nonfood) and mode (VR vs RL) as independent variables and arousal as the dependent variable was performed. No significant main effect of stimulus type was found (*F*_1,158_=0.826, *P*=.37, partial η^2^=0.005). No main effect of mode was found (*F*_1,158_=0.187, *P*=.67, partial η^2^=0.001). The interaction between stimulus type and mode was not significant (*F*_1,158_=0.119, *P*=.73, partial η^2^=0.001). R^2^ of the complete model was 0.007 (adjusted R^2^=–0.012).

### Effect of Mode (VR vs RL) on Food Attractiveness and Desire to Eat

To test whether food cravings produced by VR food cues are similar to those produced by real food, ANOVA with mode (VR vs RL) as the independent variable and chocolate attractiveness (answer to “How appealing was the chocolate you saw in the game?”) as the dependent variable was performed. Note that we only used half of the data set for subsequent analyses because only half of the participants were exposed to food cues. No main effect of mode was found (*F*_1,79_=1.122, *P*=.29, partial η^2^=0.014). R^2^ of the model was 0.014 (adjusted R^2^=0.002).

ANOVA with mode (VR vs RL) as the independent variable and the desire to eat chocolate (answer to “How much did you feel like eating the chocolate?”) as the dependent variable was performed. No main effect of mode was found (*F*_1,79_=2.607, *P*=.11, partial η^2^=0.033). R^2^ of the model was 0.033 (adjusted R^2^=0.020).

### Exploratory Analysis

**Figure 2:**
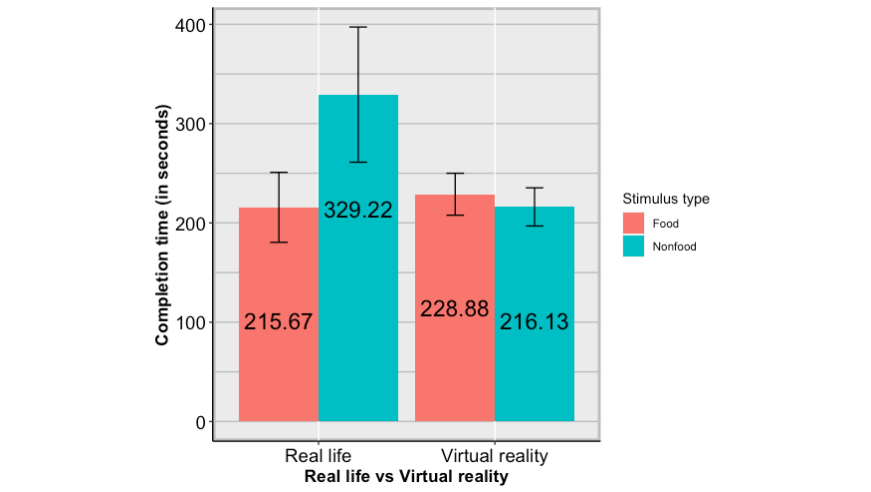
The interaction between stimulus type and mode on the completion time.

In this study, we also measured the completion time of the puzzle game. To test whether participants spent a similar amount of time in completing the puzzle game between VR and RL contexts, ANOVA with stimulus type (food vs nonfood) and mode (VR vs RL) as independent variables and completion time as the dependent variable was performed. As shown in Figure 2, a significant main effect of stimulus type was found (*F*_1,158_=6.040, *P*=.02, partial η^2^=0.037). The completion time was longer in the nonfood condition (M=274.088, SD 168.764) compared to the food condition (M=222.110, SD 92.864). A main effect of mode was found (*F*_1,158_=5.930, *P*=.02, partial η^2^=0.036). The completion time (seconds) was longer in the RL condition (M=271.759, SD 179.864) than in the VR condition (M=222.582, SD 62.765). The interaction between stimulus type and mode was also significant (*F*_1,158_=9.482, *P*=.002, partial η^2^=0.057). Simple contrasts revealed that when in RL mode, the completion time was longer in the nonfood condition (*F*_1,158_=15.713, *P*<.001; M=329.220, SD 215.684) than in the food condition (M=215.667, SD 113.105). However, in VR mode, there was no significant difference in the completion time between the 2 conditions (M_nonfood_=216.128, SD 59.299 vs M_food_=228.875, SD 66.109; *F*_1,158_=0.188, *P*=.67). Additionally, simple contrasts revealed that when the stimulus type was nonfood, the completion time was longer in the RL condition (*F*_1,158_=15.016, *P*<.001; M=329.220, SD 215.684) than in the VR condition (M=216.128, SD 52.299). However, when the stimulus type was food, there was no significant difference in the completion time between the 2 conditions (M_VR_=228.875, SD 66.109 vs M_RL_=215.667, SD 113.105; *F*_1,158_=0.210, *P*=.65). R^2^ of the complete model was 0.120 (adjusted R^2^=0.104).

## Discussion

### Principal Results

Due to the rising popularity of VR in our daily life, it is necessary to better understand how VR food cues affect consumers’ eating behavior (eg, food intake). However, prior research has mainly focused on the impact of VR food cues on food cravings [7,12,20,21]. In this study, we examined the effect of interaction with food cues (vs nonfood cues) on subsequent food intake in both VR and RL contexts, with the aim to replicate the pre-exposure effect and to extend this effect to VR contexts.

Unexpectedly, we found that the main effect of mode (VR vs RL) on food intake is significant. Specifically, we found a higher food intake after both VR conditions (puzzle game in VR with either food or nonfood cues) than after RL conditions. A possible explanation could be that playing a game in VR is arousing and that arousal leads to increased food intake. There is ample evidence that arousal may lead to increased food intake [28]. However, it should be noted that in this study, there was no significant difference in self-reported arousal between VR and RL conditions. In contrast, there is also some evidence that playing games may decrease food cravings [29], possibly because playing games distracts from feelings of hunger and craving. Given the unexpected nature of this finding, it is important that this be replicated.

We did not replicate the pre-exposure effect in the full sample. In addition, there was no interaction effect between stimulus type (food vs nonfood) and mode (VR vs RL) on food intake in the full sample. Prior studies have shown that pre-exposure effects are in some instances specific to males [15] or females [6]. Therefore, we examined whether our results were contingent on gender. In the male sample, 2-way ANOVA did not reveal any significant effects. In the female sample, however, we found that exposure to both food cues (puzzle game with food cues with either VR or RL) decreased food intake than exposure to nonfood cues. This is in line with one of the prior studies on the pre-exposure effect [6]. We found that females reported a higher chocolate preference (ie, liking for chocolates) than males, which suggests that females may be more tempted by chocolates than males; liking for chocolates was M_female_=6.000 (SD 1.147) versus M_male_=5.364 (SD 1.382), with *F*_1,160_=8.792 and *P*=.003. Consequently, given that tempting chocolates may induce a behavioral conflict between the desire to eat and the completion of a food-related task among females, we observed the pre-exposure effect. In contrast, chocolates may not have been sufficiently tempting for males, therefore resulting in a lack of behavioral conflict and activation of control processes in males.

Moreover, we found that emotional and behavioral responses (eg, valence, arousal, entertainment, and difficulty) induced by VR cues are similar to those generated by RL cues. This suggests that there were no additional confounders between the conditions. In addition, this study showed that exposure to VR food cues elicits similar food evaluations (ie, attractiveness and a desire to eat) compared to exposure to RL food cues. Given that the appeal of food (measured in this study) was similar to food cravings (measured in prior studies), this study does not provide evidence that VR food cues induce weaker food cravings compared to RL food cues [7,22]. In the study by Ledoux et al [7], the impact of VR food cues (vs RL food cues) on food cravings was examined among nondieting women. This study conducted this investigation with a more general sample including both males and females, as well as both dieting and nondieting individuals. However, a more general sample was also used in the study by van der Waal et al [22]. We speculate that individual differences (eg, gender, age, and eating/dieting habits) may moderate the effect of VR food cues on food cravings. This needs further investigation with a more diverse sample before firm conclusions can be drawn.

### Limitations and Future Research

Although this study provides useful insights into the impact of VR cues on food intake, 3 limitations should be considered. First, in this research, we found that manipulation affects the completion (exposure) time of puzzle games. Specifically, participants spent more time completing puzzle games in nonfood or RL conditions as compared to food or VR conditions. We tried to replicate the pre-exposure effect in RL contexts; however, the completion time was longer in the nonfood condition (around 5.5 minutes) than in the food condition (around 3.5 minutes). Prior research on the pre-exposure effect used a consumer knowledge task [4], a puzzle-solving task [3], or a word formation task [14]. In the puzzle-solving task (a total of 8 puzzles), participants were instructed to solve each puzzle within a time limit (40 seconds) in both food and nonfood conditions, indicating that the total exposure time (around 5.5 minutes) was the same between the two conditions. In addition, in the word formation task, both groups (candies vs foams) were instructed to complete the task in an allocated time of 4 minutes [14]. In other words, prior studies have revealed that an exposure period of 4-6 minutes may be necessary for the pre-exposure effect to occur after exposure to RL food cues. In addition, building on prior studies on the pre-exposure effect, there was no difference in the completion (exposure) time between food and nonfood conditions. In this study, therefore, we speculate that the time difference between the 2 conditions (food vs nonfood) and the insufficient exposure time in the food condition may account for the inability to replicate the pre-exposure effect in RL contexts. To rule out the impact of the completion (exposure) time, follow-up studies should allocate a fixed time duration in the exposure phase.

Second, this research used the pre-exposure paradigm (eg, a puzzle game and a taste test) to examine the effect of VR cues on food intake. Both tasks are more specific to laboratory contexts. Until now, we are not clear whether the pre-exposure procedure still works well outside laboratory contexts. Given the unlimited possibility of creating various eating environments with HMD-VR, future research could examine how VR affects food intake in different situations. Prior research on VR food cues focused on some daily environments, such as living rooms, kitchens, and restaurants. For instance, there was no significant difference in food intake between the restaurant scene and the blank scene in VR environments [23]. However, that study did not introduce the pre-exposure paradigm. Consumers’ eating behaviors may be different between laboratory and RL contexts [23]. In a laboratory context, participants may feel that their eating behaviors are being observed and then behave differently as compared to their typical eating habits in RL environments [30]. VR can be used to recreate any eating environment similar to RL situations; thus, to generalize the pre-exposure effect, future studies could examine the pre-exposure procedure in VR cinema or a VR cafeteria.

Third, in this study, we exclusively used confectionery food products when manipulating pre-exposure (ie, chocolate) and when measuring food intake (ie, grams of M&M’s). Both products belong to the same food category, which warrants caution with generalizing our results across all food products. Concretely, individual differences in perceptions of how appealing the stimulus food was, as well as strong preferences in particular brands of confectionery food products, could have affected food intake (at least) on the individual level. To rule out any potential product-specific bias, future studies could consider incorporating different or various food categories to manipulate pre-exposure as well as to measure food intake.

### Theoretical and Practical Implications

Our findings contribute to 2 streams of literature: the pre-exposure procedure and the responses to VR food cues. Although prior research on the pre-exposure procedure has studied how food cues versus nonfood cues affect the subsequent intake of tempting foods in RL contexts [3,4,15], our research goes further by also exploring the role of VR in the pre-exposure procedure. Despite not replicating the pre-exposure effect in VR, our study did show that exposure to cues in VR, generally, leads to higher food intake than exposure to cues in RL. This seems particularly important for health practitioners developing (food-related) VR interventions, who should consider that the mere act of being in VR may elevate food intake, at least directly after the experience.

Furthermore, our research extends the VR food cues literature by examining the impact of VR on actual food intake, instead of food cravings, considering that actual food intake is critical to understanding whether VR food cues increase or decrease subsequent intake of tempting foods in more naturalistic settings. In addition, in line with prior research on the effects of VR food cues on eating behavior–related outcomes [9,10], the findings of this research offer additional insights into the similarity between VR food cues and RL food cues.

### Conclusion

Overall, in this study, we were unable to replicate the exposure effect in our complete sample. Subgroup analyses, however, showed that for women, exposure to food cues (either in VR or in RL) does reduce food intake, indicating that a VR pre-exposure procedure may effectively be applied exclusively for women. Moreover, we found that exposure to cues in VR (either food or nonfood) results in a higher overall food intake as compared to exposure to cues in a similar RL setting. Finally, we demonstrated that VR and RL cues elicit similar emotional responses (eg, arousal and valence).

## Appendix

Multimedia Appendix 1 Chocolate tangram (puzzle game).

Multimedia Appendix 2 CONSORT-eHEALTH checklist (V 1.6.1).

## Abbreviations

BMI: body mass index
HMD-VR: head-mounted display virtual reality
RL: real life
SAM: Self-Assessment Manikin
VAS: visual analog scale
VR: virtual reality

## References

[1] van der Laan LN, de Ridder DTD, Viergever MA, Smeets PAM (2011). The first taste is always with the eyes: a meta-analysis on the neural correlates of processing visual food cues. Neuroimage.

[2] Chandon P, Wansink B (2012). Does food marketing need to make us fat? A review and solutions. Nutr Rev.

[3] Goddyn H, Dewitte S (2017). Handling tempting food in a non-consummatory context reduces subsequent consumption of other tempting food: an extension beyond sweet snacks. Food Qual Prefer.

[4] Geyskens K, Dewitte S, Pandelaere M, Warlop L (2008). Tempt me just a little bit more: the effect of prior food temptation actionability on goal activation and consumption. J Consum Res.

[5] Duh H, Grubliauskiene A, Dewitte S (2016). Pre-exposure to food temptation reduces subsequent consumption: a test of the procedure with a South-African sample. Appetite.

[6] de Boer C, de Ridder D, de Vet E, Grubliauskiene A, Dewitte S (2015). Towards a behavioral vaccine: exposure to accessible temptation when self-regulation is endorsed enhances future resistance to similar temptations in children. Appl Psychol Health Well Being.

[7] Ledoux T, Nguyen AS, Bakos-Block C, Bordnick P (2013). Using virtual reality to study food cravings. Appetite.

[8] Kang H, Shin J, Ponto K (2020). How 3D virtual reality stores can shape consumer purchase decisions: the roles of informativeness and playfulness. J Interact Mark.

[9] Diemer J, Alpers GW, Peperkorn HM, Shiban Y, Mühlberger A (2015). The impact of perception and presence on emotional reactions: a review of research in virtual reality. Front Psychol.

[10] Gorini A, Griez E, Petrova A, Riva G (2010). Assessment of the emotional responses produced by exposure to real food, virtual food and photographs of food in patients affected by eating disorders. Ann Gen Psychiatry.

[11] Siegrist M, Ung C, Zank M, Marinello M, Kunz A, Hartmann C, Menozzi M (2019). Consumers' food selection behaviors in three-dimensional (3D) virtual reality. Food Res Int.

[12] Ferrer-Garcia M, Gutierrez-Maldonado J, Treasure J, Vilalta-Abella F (2015). Craving for food in virtual reality scenarios in non-clinical sample: analysis of its relationship with body mass index and eating disorder symptoms. Eur Eat Disord Rev.

[13] Flavián C, Ibáñez-Sánchez S, Orús C (2019). The impact of virtual, augmented and mixed reality technologies on the customer experience. J Bus Res.

[14] Stamos A, Goddyn H, Andronikidis A, Dewitte S (2018). Pre-exposure to tempting food reduces subsequent snack consumption in healthy-weight but not in obese-weight individuals. Front Psychol.

[15] Grubliauskiene A, Dewitte S (2014). Temptation in the background: non-consummatory exposure to food temptation enhances self-regulation in boys but not in girls. Front Psychol.

[16] Dewitte S, Bruyneel S, Geyskens K (2009). Self-regulating enhances self-regulation in subsequent consumer decisions involving similar response conflicts. J Consum Res.

[17] Florack A, Haasova S, Hirschauer S, Serfas BG (2018). Playing with food: the effects of food pre-exposure on consumption in young children. Physiol Behav.

[18] Ung C, Menozzi M, Hartmann C, Siegrist M (2018). Innovations in consumer research: the virtual food buffet. Food Qual Prefer.

[19] van Herpen Erica, van den Broek Eva, van Trijp Hans C M, Yu T (2016). Can a virtual supermarket bring realism into the lab? Comparing shopping behavior using virtual and pictorial store representations to behavior in a physical store. Appetite.

[20] Ferrer-Garcia M, Pla-Sanjuanelo J, Dakanalis A, Vilalta-Abella F, Riva G, Fernandez-Aranda F, Sánchez I, Ribas-Sabaté J, Andreu-Gracia A, Escandón-Nagel N, Gomez-Tricio O, Tena V, Gutiérrez-Maldonado J (2017). Eating behavior style predicts craving and anxiety experienced in food-related virtual environments by patients with eating disorders and healthy controls. Appetite.

[21] Ferrer-García M, Gutiérrez-Maldonado J, Pla J (2013). Cue-elicited anxiety and craving for food using virtual reality scenarios. Stud Health Technol Inform.

[22] van der Waal NE, Janssen L, Antheunis M, Culleton E, van der Laan LN (2021). The appeal of virtual chocolate: a systematic comparison of psychological and physiological food cue responses to virtual and real food. Food Qual Prefer.

[23] Oliver JH, Hollis JH (2021). Virtual reality as a tool to study the influence of the eating environment on eating behavior: a feasibility study. Foods.

[24] van Berlo ZMC, van Reijmersdal EA, Smit EG, van der Laan LN (2020). Inside advertising: the role of presence in the processing of branded VR content. Augmented Reality and Virtual Reality.

[25] van Berlo ZM, van Reijmersdal EA, Smit E, van der Laan LN (2021). Brands in virtual reality games: affective processes within computer-mediated consumer experiences. J Bus Res.

[26] Martí-Parreño J, Aldás-Manzano J, Currás-Pérez R, Sánchez-García I (2012). Factors contributing brand attitude in advergames: entertainment and irritation. J Brand Manag.

[27] Bradley MM, Lang PJ (1994). Measuring emotion: the Self-Assessment Manikin and the semantic differential. J Behav Ther Exp Psychiatry.

[28] Stroebele N, de Castro JM (2006). Influence of physiological and subjective arousal on food intake in humans. Nutrition.

[29] Skorka-Brown J, Andrade J, May J (2014). Playing 'Tetris' reduces the strength, frequency and vividness of naturally occurring cravings. Appetite.

[30] Robinson E, Hardman CA, Halford JCG, Jones A (2015). Eating under observation: a systematic review and meta-analysis of the effect that heightened awareness of observation has on laboratory measured energy intake. Am J Clin Nutr.

