# Supplementary figures and images for: Development and Evaluation of a Virtual Reality Puzzle Game to Decrease Food Intake: Randomized Controlled Trial
Source: JMIR Serious Games. 2022 Feb 3;10(1):e31747. doi: 10.2196/31747 (PMC8855293; doi:10.2196/31747)

## Slide 1
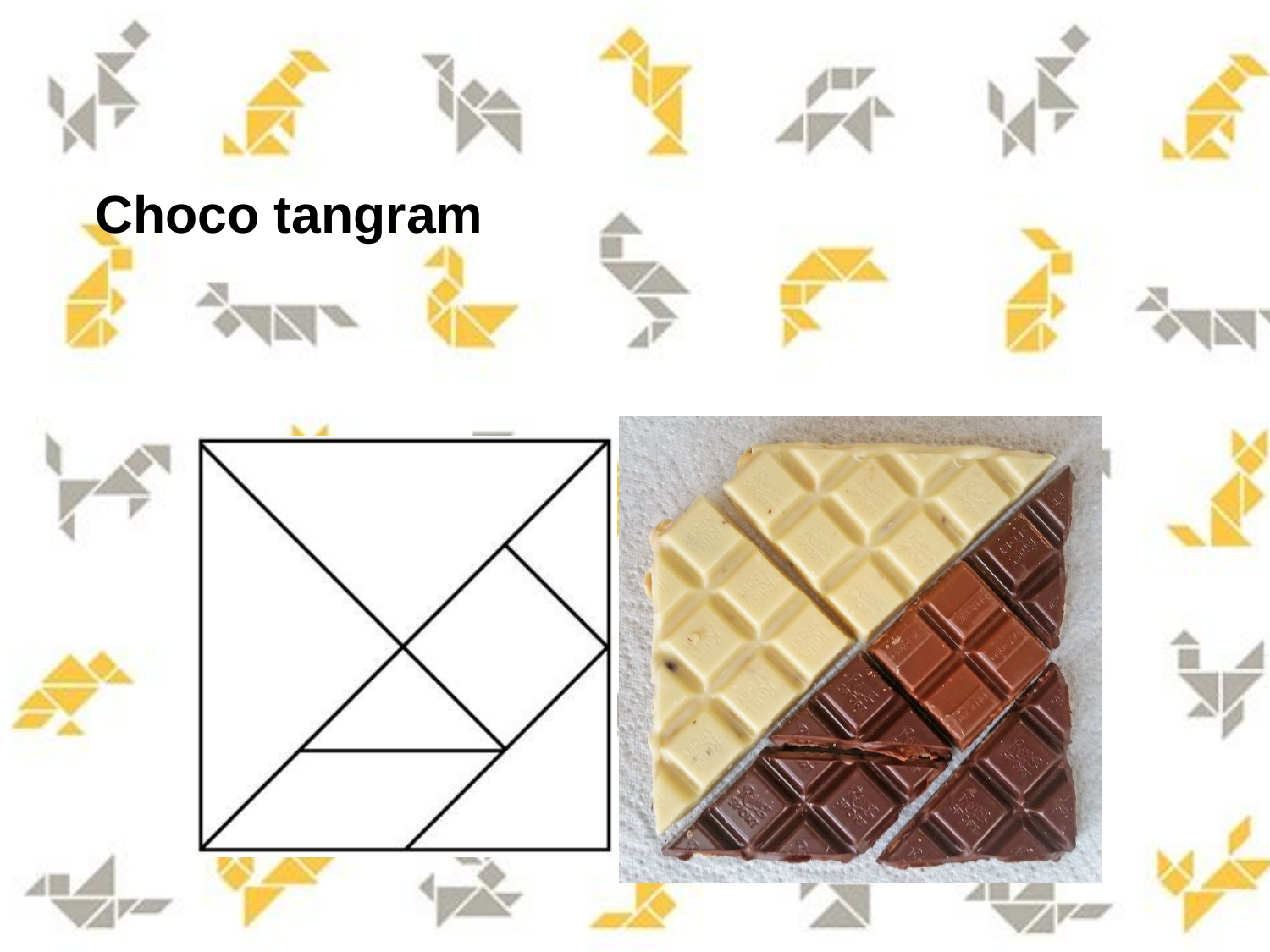

# Choco tangram
1

## Slide 2
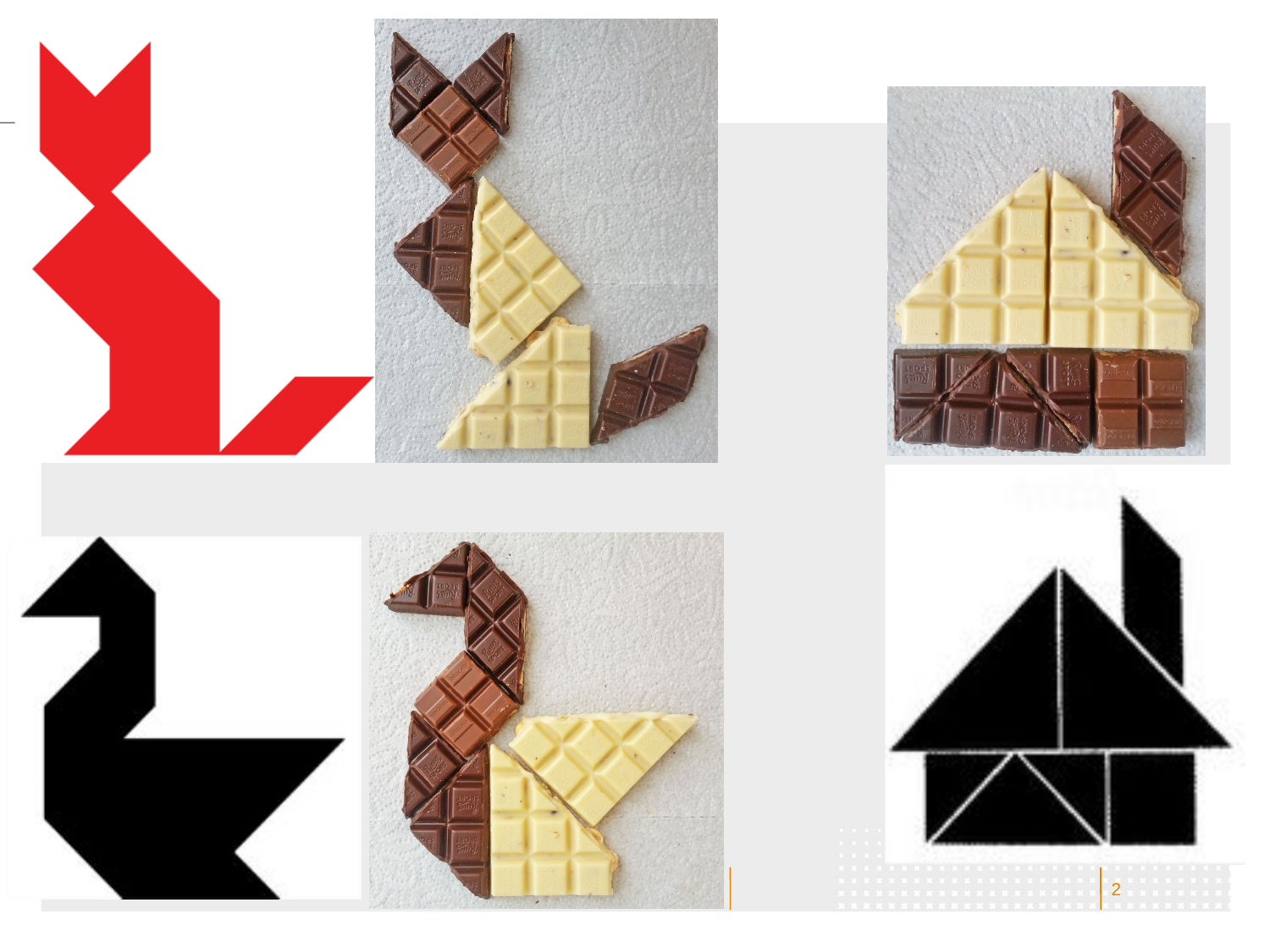

#
2

Supplement: Multimedia Appendix 1 [file games_v10i1e31747_app1.pptx]
